# Changes in Opioid Use After Florida’s Restriction Law for Acute Pain Prescriptions

**DOI:** 10.1001/jamanetworkopen.2020.0234

**Published:** 2020-02-28

**Authors:** Juan M. Hincapie-Castillo, Amie Goodin, Marie-Christin Possinger, Silken A. Usmani, Scott Martin Vouri

**Affiliations:** 1 Center for Drug Evaluation and Safety, University of Florida, Gainesville; 2 Department of Pharmaceutical Outcomes and Policy, University of Florida, Gainesville; 3 Pain Research and Intervention Center of Excellence, University of Florida, Gainesville; 4 UF Health Physicians, Gainesville, Florida

## Abstract

This quality improvement study assesses the number of new opioid users and the number of units dispensed per prescription before and after implementation of Florida’s 2018 restriction law on opioid prescribing.

## Introduction

Several US states have enacted laws to limit either opioid prescription days’ supply or morphine milligram equivalents for the treatment of acute pain.^1^ On July 1, 2018, Florida implemented House Bill 21 (HB21), which limits the days’ supply of Schedule II opioids to 3 days for acute pain prescriptions.^2^ Prescribers can extend to a 7-day supply if they document an exception. This quality improvement study aims to assess the outcomes associated with Florida’s restriction law on opioid prescribing by evaluating the number of new opioid users and the number of units dispensed per prescription before and after the policy change.

## Methods

This study was approved by the institutional review board at the University of Florida and was exempted from informed consent requirements because data were deidentified. This study follows the Standards for Quality Improvement Reporting Excellence (SQUIRE) reporting guideline.

We analyzed pharmacy prescription claims for opioids dispensed from January 2015 through March 2019 from a single health plan serving more than 45 000 employees of a large Florida employer. We included single-entity and combination products of oral opioid formulations. Patients were considered opioid naive if there were no other opioid prescription claims in the preceding 180 days. Patients could be counted again as new users if there were subsequent opioid claims that occurred 180 days or more apart. Outcome measures included new opioid users per 1000 health plan enrollees per month and mean days’ supply of the opioid prescription per month.

We conducted analyses with an interrupted time series method, fitting generalized least squares linear models with an autoregressive–moving average correlation structure. We used R statistical software version 3.6.1 (R Project for Statistical Computing) for our calculations. A 2-sided *P* < .05 was considered statistically significant in evaluating the model coefficients for time effect and level and trend changes resulting from the policy interruption. Data analysis was conducted from April 2019 to August 2019.

## Results

There were 54 409 individual opioid prescriptions dispensed to plan enrollees during the study period. After applying the inclusion criteria, there were a total of 8375 patients with 10 583 unique opioid starts.

**Figure 1.**
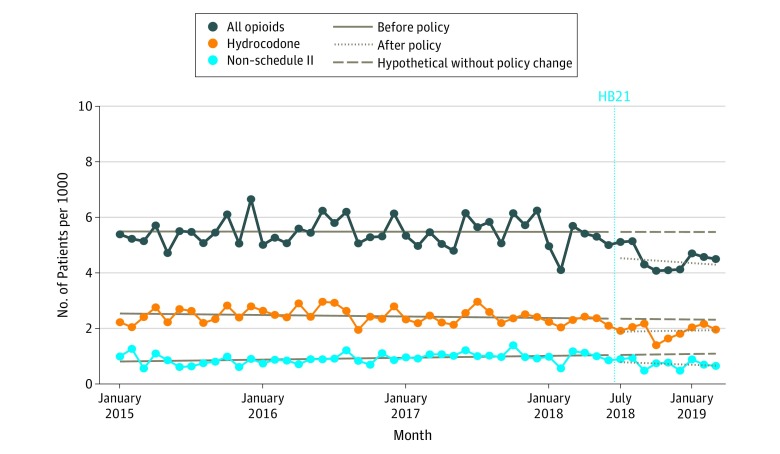
Trends in Number of New Opioid Users per Month, January 2015 to March 2019 Vertical dashed line indicates implementation of Florida House Bill 21 (HB21).

Before HB21, 5.5 patients per 1000 enrollees per month began opioid use; there was a significant decrease in incidence immediately after the law was implemented to 4.6 new users per 1000 enrollees per month (decrease, −0.92 new user per 1000 enrollees per month; 95% CI, −1.53 to −0.31 new users per 1000 enrollees per month; *P* = .005) (Figure 1). Implementation of the law was also associated with an immediate decrease in use of hydrocodone (decrease, −0.48 new user per 1000 enrollees per month; 95% CI, −0.92 to −0.03 new user per 1000 enrollees per month) and use of non–Schedule II opioids (decrease, −0.24 new user per 1000 enrollees per month; 95% CI, −0.48 to 0.001 new user per 1000 enrollees per month).

**Figure 2.**
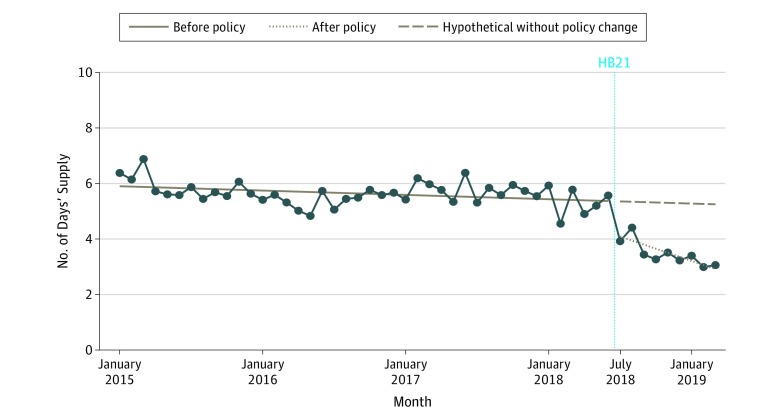
Trend in Number of Days’ Supply per Opioid Prescription per Month, January 2015 to March 2019 Vertical dashed line indicates implementation of Florida House Bill 21 (HB21).

The mean (SD) days’ supply of opioids was 5.4 (6.5) days per prescription before the implementation of HB21 (Figure 2). Implementation of the law was associated with a significant immediate reduction to 4.2 days per prescription (decrease, −1.13 days per prescription; 95% CI, −1.78 to −0.48 days per prescription; *P* = .001) and a continuous decreasing trend over the following 8 months (decrease, −0.13 day per month; 95% CI, −0.24 to −0.02 day per month). The mean (SD) days’ supply of opioids was 3 (1.5) days by the end of the study period.

## Discussion

These findings suggest that implementation of HB 21 in Florida was associated with decreased opioid use and with changes in initial prescribing decisions. Our findings are more pronounced than those reported in analyses of laws in other states,^3,4,5^ but it should be noted that Florida’s 3-day restriction law is more stringent than other evaluated restriction laws. Generalizability of these findings is limited because the privately insured population was composed of patients who were younger and healthier than the typical opioid initiator. Future studies are needed to evaluate the downstream outcomes of these laws on use for appropriate and inappropriate treatment alternatives, as well as changes in nonmedical opioid use. Because there is a federal initiative to implement prescription opioid restrictions for acute pain, additional studies of these state laws are needed to inform policy makers.^6^
